# MQTT-Based Architecture for Real-Time Data Collection and Anomaly Detection in Smart Livestock Housing

**DOI:** 10.3390/s25237186

**Published:** 2025-11-25

**Authors:** Kyeong Il Ko, Meong Hun Lee

**Affiliations:** Department of Smart Agriculture Program, Sunchon National University, Suncheon-si 31031, Republic of Korea; 1245024@s.scnu.ac.kr

**Keywords:** smart livestock housing, MQTT, anomaly detection, GRU, QoS, low-latency monitoring

## Abstract

This study designed a message queuing telemetry transport (MQTT)-based communication framework to acquire environmental data with stable, low-latency response (soft real-time capability) and detect anomalies in smart livestock housing. We validated the performance of the proposed framework using actual sensor data. It comprises environmental sensor nodes, a Mosquitto MQTT broker, and a GRU-based anomaly detection model, with data transmission via a WiFi-based network. Comparing quality of service (QoS) levels, the QoS 1 configuration demonstrated the most stable performance, with an average latency of ~150 ms, a data collection rate ≥ 99%, and a packet loss rate ≤ 0.5%. In the sensor node expansion experiment, responsiveness (≤200 ms) persisted for 10–15 nodes, whereas latency increased to 238.7 ms for 20 or more nodes. The GRU model proved suitable for low-latency analysis, achieving 97.5% accuracy, an F1-score of 0.972, and 18.5 ms/sample inference latency. In the integrated experiment, we recorded an average end-to-end latency of 185.4 ms, a data retention rate of 98.9%, processing throughput of 5.39 samples/s, and system uptime of 99.6%. These findings demonstrate that combining QoS 1-based lightweight MQTT communication with the GRU model ensures stable system response and low-latency operation (soft real-time capability) in monitoring livestock housing environments, achieving an average end-to-end latency of 185.4 ms.

## 1. Introduction

Globally, the livestock industry is essential for food security, economic growth, and sustaining rural livelihoods. According to the Food and Agriculture Organization, livestock farming accounts for approximately 40% of global agricultural production and is directly linked to the livelihoods of over one billion individuals [1]. Traditional livestock farming methods have exhibited sustainability constraints owing to threats such as climate change, infectious diseases, and declining productivity. Consequently, smart livestock farming has garnered global attention as a strategy to address these challenges and maintain competitiveness in the industry.

This system acquires and analyzes continuous data on livestock growth, environmental conditions, and production using the Internet of Things (IoT), artificial intelligence, sensor technology, and information and communication technology (e.g., cloud computing) to optimize feeding management and decision-making. In particular, the implementation of smart livestock farming technology is actively promoted by the EU, USA, and Australia to enhance livestock welfare, comply with environmental regulations, and improve production efficiency. It is also considered a key element of digital transformation in the global livestock sector [2,3].

A core technology in smart livestock farming is a monitoring and control system for livestock housing environments requiring rapid, stable response. Temperature, humidity, carbon dioxide (CO_2_), and ammonia (NH_3_) levels within these environments directly influence animal health and productivity. Enclosed livestock housing is highly sensitive to environmental fluctuations and therefore requires efficient monitoring. For example, a temperature and humidity imbalance may reduce feed intake, increase respiratory disease incidence, and elevate pig mortality, thus diminishing productivity and causing economic losses for farms [4].

Conventional environmental monitoring systems for livestock housing rely primarily on RS-485 or legacy communication methods, which restrict scalability, maintenance, and responsiveness. These systems exhibit low management efficiency in large-scale operations owing to complex sensor architectures and poor device interoperability. Moreover, many systems merely store collected data or require manual analysis, hindering prompt detection and early anomaly responses. This further highlights the need for a proactive environmental sensitivity system in livestock housing [5].

To satisfy low-latency and scalability requirements in multisensor livestock environments, message queuing telemetry transport (MQTT), a lightweight messaging protocol, has emerged as a promising alternative [6]. Conventional systems typically use RS-485 wired communication [7], Modbus RTU [8], or WiFi-based HTTP methods. The RS-485 and Modbus approaches involve complex wiring and architectures and lose flexibility as node counts grow. Meanwhile, the HTTP method has limited capability in maintaining continuous connections owing to its request-response framework; this structurally limits continuous sensor data transmission [9].

By contrast, MQTT employs an asynchronous publish/subscribe model within a client-broker-subscriber architecture. This architecture enables data collection and processing in dense sensor networks. It supports reliable, scalable low-latency communication via quality of service (QoS) settings, message retention (Retain), and reduced overhead. Therefore, MQTT has been applied in fields such as smart agriculture, industrial IoT, and energy monitoring, and has been identified as a highly suitable communication protocol for smart livestock housing systems [6].

In this study, we designed a system for environmental data collection and anomaly detection using an MQTT communication architecture applied to data from livestock housing environments, quantitatively evaluating both communication and ML analysis performance. We measured the MQTT broker’s performance with respect to sensor count, message transmission interval, and QoS settings, and compared the ML models’ accuracy and processing time to assess low-latency performance and intelligent response in smart livestock housing environments. Ultimately, the system was implemented and evaluated for continuous data collection and anomaly detection by using actual livestock housing environments.

This study aimed to implement a system for continuous environmental data collection and anomaly detection using an MQTT communication architecture based on data acquired from actual livestock housing environments. The performance of the proposed system was evaluated, and the major contributions of this study are as follows:

(1) An MQTT-based communication system was designed using actual environmental data, and its performance (message latency and transmission stability) was experimentally analyzed with respect to sensor count, transmission interval, and QoS settings;

(2) ML models for detecting environmental anomalies were applied, comparing various algorithms in terms of predictive accuracy and suitability for low-latency inference;

(3) The overall response time was assessed by integrating MQTT communication latency and ML inference time to evaluate the smart livestock housing system’s stable, low-latency applicability;

(4) A foundation for future responsive control systems and smart livestock housing automation is established by empirically demonstrating integration of a lightweight, communication-based IoT system with an intelligent environmental analysis model for the livestock sector.

## 2. Related Research

### 2.1. Livestock Housing Environment Monitoring and Conventional Communication Technologies

In smart livestock housing environments, precise monitoring of key environmental variables (e.g., temperature, humidity, CO_2_, and NH_3_) is crucial for maintaining livestock health and productivity. Various communication technologies have been introduced in the livestock industry; however, conventional approaches exhibit structural limitations in modern smart housing, which demands high-frequency, continuous data collection from multiple sensors. A comparative summary of representative communication technologies applied in livestock housing environments is provided in Table 1.

RS-485-based wired communication is a serial method extensively employed in industrial automation over an extended period [7]. They are highly resistant to noise while supporting long-distance transmission. In livestock housing, this technology has been adopted to provide stable communication between temperature/humidity sensors and ventilation systems; RS-485 serves as the principal communication method in environmental monitoring systems deployed in enclosed pig farms in China [10]. However, it requires physical wiring, which complicates the architecture as sensor nodes increase, thus raising installation and maintenance burdens. Moreover, flexible scalability diminishes in the master–slave architecture because devices must be linearly connected, and a single failure may compromise overall system reliability [11].

Modbus RTU over RS-485 is a request–response protocol commonly implemented in early livestock housing IoT systems because of its broad compatibility with commercial sensors and control units. Its simple frame structure simplifies implementation, and it has received favorable evaluations for data accuracy and stability [8,12]. Nevertheless, as exchanges occur only at the master’s request, asynchronous message transmission or prompt event-based processing becomes challenging, while sequential processing induces communication bottlenecks as sensor numbers grow [13]. These drawbacks limit low-latency capabilities in livestock housing environments requiring high-speed data collection.

WiFi-based-based HTTP communication has been adopted in some smart-farm systems owing to its straightforward implementation in wireless networks and seamless integration with cloud-based remote monitoring systems. In fact, cases exist in which the HTTP-based data transmission/reception architecture was applied in poultry farms and small facilities to achieve low-cost configuration and enhanced user accessibility [14]. However, HTTP encounters challenges in maintaining continuous connections due to its inherent request-response architecture; connection delays and data transmission omissions are common. Moreover, HTTP is unsuitable for simultaneously streaming high-frequency continuous data or processing inputs from numerous sensors, and its high power consumption is also unfavorable in battery-powered sensor-network environments.

Although conventional communication technologies demonstrate a certain level of stability and applicability, they cannot fully satisfy the scalability, low-latency performance, maintenance efficiency, and asynchronous data processing requirements of modern smart livestock housing environments. To address these limitations, this study introduces an MQTT communication protocol characterized by a lightweight architecture and an asynchronous publish/subscribe method and quantitatively analyzes its performance based on actual environmental data.

### 2.2. Research on MQTT-Based Smart Livestock Farming Applications

For IoT-based smart livestock housing systems, demand is growing for a communication infrastructure that reliably collects high-frequency data from multiple sensors and processes them without delay. MQTT—a lightweight message communication protocol—has quickly been adopted in diverse agricultural and livestock applications to address these requirements. Employing a broker-based publish/subscribe architecture, MQTT offers low-latency performance and scalability in multisensor environments due to low bandwidth demand, high reliability, configurable QoS levels, and Retain. Unlike conventional request–response protocols, MQTT permits asynchronous reception when a client publishes data to the broker, rendering it especially suitable for smart livestock housing environments characterized by substantial data flows and critical rapid responses.

Provolo et al. utilized this architecture to continuously collect temperature, humidity, NH_3_, and CO_2_ data from enclosed pig farms and implemented a platform to control an automatic ventilation system. This system demonstrated lower latency and higher packet transmission reliability compared with an HTTP-based environment, with insignificant performance degradation as the number of sensors increased [15].

Similarly, Dineva et al. constructed an MQTT-based multichannel sensor platform to continuously analyze NH_3_ concentration and gas mixing, proposing a system that integrates data monitoring with cloud servers [16].

He et al. constructed an MQTT cluster system within a suckling piglet breeding environment using an electronic sow feeder. The system was designed to collect data reliably even in high-concurrency environments by employing a ZooKeeper-based MQTT server cluster architecture [17].

Finally, Has et al. subsequently investigated improvements in communication efficiency and security by combining MQTT with encryption and data compression technologies in agricultural IoT data management, illustrating its potential for livestock data transmission in bandwidth-limited or security-critical environments [18].

Previous studies empirically determined that MQTT is suitable for smart livestock housing; however, they primarily concentrated on communication architecture design and system implementation. Few studies quantitatively analyzed performance in relation to QoS settings, sensor count, and message transmission cycles. Moreover, most research efforts have focused on visualizing or storing environmental data, with limited success in promptly detecting anomalies or integrating control algorithms.

This study constructed an MQTT-based communication architecture using actual environmental data from prior research, quantitatively evaluating transmission performance under various communication conditions. Additionally, it analyzed overall system response through integration with ML-based anomaly-detection models to demonstrate the feasibility of implementing low-latency monitoring and intelligent control architectures in smart livestock housing.

### 2.3. ML Applications for Detecting Environmental Anomalies

Early detection of anomalies in environmental variables in smart livestock housing is recognized as a crucial technology for safeguarding livestock health and preventing productivity loss. Accordingly, diverse ML-based analytical techniques have been introduced to detect anomalies in environmental sensor data, offering alternatives to traditional threshold-based methods.

Chen et al. [19] proposed an anomaly detection method for wireless sensor networks based on an improved Isolation Forest algorithm. Their approach addresses the issues of computational efficiency and detection accuracy found in traditional methods, proving effective for processing environmental sensor data in monitoring systems.

Zang et al. [20] established a prediction model for carbon dioxide (CO_2_) concentration in pig houses using deep learning techniques. Their study demonstrated that deep learning-based models can effectively predict environmental fluctuations and provide support for precise environmental control in livestock housing.

Park et al. [21] applied deep learning techniques to detect anomalies in the operating equipment of livestock farms. By analyzing operational data, they successfully identified irregular patterns, highlighting the effectiveness of deep learning algorithms for maintaining stable smart livestock production environments.

These studies collectively demonstrated the effectiveness of ML-based anomaly detection algorithms. Few studies have addressed integration with communication architecture, linkage to responsive systems, or the end-to-end performance of the entire arrangement. This study investigates these issues by. conducting MQTT-based communication performance experiments alongside an integrated analysis of ML-based anomaly detection models to quantitatively assess low-latency applicability.

## 3. System Design and Analysis Method

### 3.1. System Configuration Overview

**Figure 1:**
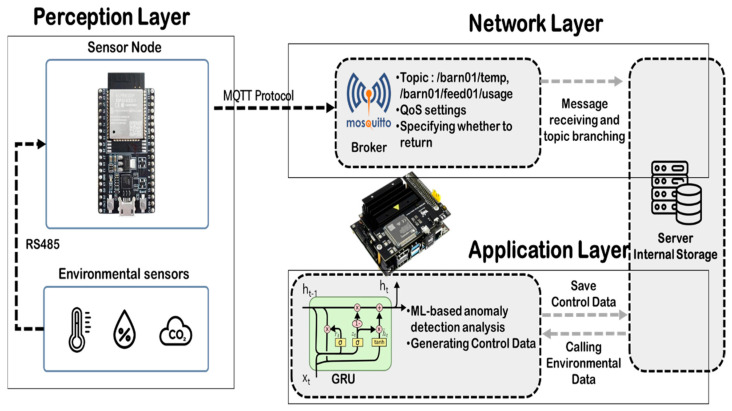
System Overview (Adapted from [22]).

The smart livestock housing data collection and analysis system designed herein efficiently transmits environmental sensor data and generates control information via anomaly analysis. The system comprises three layers: perception, network, and application (Figure 1); each functions independently while remaining interconnected.

The Perception Layer, as the physical level, collects environmental data. It consists of temperature, humidity, CO_2_, and NH_3_ sensors and feeding management systems (e.g., feeding devices). Each sensor node converts measured data into MQTT messages and transmits them to the broker via WiFi-based.

The Network Layer, focused on a Mosquitto-based MQTT broker, was implemented on the NVIDIA Jetson TX1 Edge Node (NVIDIA Corporation, Santa Clara, CA, USA) to process messages received from sensor nodes by topic. Message flow follows a hierarchical topic structure (e.g., /edv01/temp, /edv01/ammonia, and /edv01/feed01/usage) to enable clients to efficiently receive only the required data. Moreover, transmission reliability is ensured through MQTT’s QoS settings, and the last message status is preserved by the Retain flag. This design renders high flexibility, scalability, and low-latency communication while minimizing communication overhead.

The Application Layer is a logical layer responsible for process analysis and control of information derived from collected data, utilizing the GRU-based anomaly detection model for intelligent environmental assessment. It identifies abnormal environmental variable changes using an ML-based anomaly-detection algorithm and produces standardized control data. This standardized control data is designed to be consumed by an automation system. For instance, a ‘CO_2_ surge’ anomaly signal would logically trigger a ‘Ventilation Fan ON’ command, while an ‘NH_3_ surge’ could activate a ‘Manure Flushing’ command. In this study, direct transmission of control data to nodes via MQTT was not implemented; instead, the control information was stored locally. In future work, these data may integrate with control systems or external automation systems.

This layered architecture enhances maintainability and scalability by simplifying interlayer interfaces and logically segregating the flow from data collection to analysis. Notably, the MQTT broker and analysis engine were integrated into the Jetson device to enable low-latency processing and local, autonomous analysis without a cloud server, which is pivotal for ensuring system stability even under network connectivity constraints in smart livestock housing environments.

### 3.2. MQTT Communication Architecture and Broker Design

The implemented communication architecture was designed to continuously collect environmental data with stable, low-latency transmission from multiple sensor nodes and detect anomalies by linking to the analysis module. MQTT, a lightweight messaging protocol, was adopted for transmission. The architecture ensures efficient data flow and stable connections, even in livestock housing environments with numerous sensors and short transmission cycles.

Sensor nodes based on the ESP32 microcontroller interface with temperature, humidity, CO_2_, and NH_3_ sensors. Each node publishes messages via the Mosquitto MQTT broker installed on the Jetson TX1 over a WiFi-based network. The JSON-formatted messages contain unique device identifiers, measurement time, collection cycles, alive status, and measured values.

The Jetson TX1 comprises a high-performance edge-computing node that consolidates broker, analysis, and storage functions, surpassing the role of a conventional broker server. Eclipse Mosquitto (version 2.0.15) is an open-source software that served as the MQTT broker; its key settings are detailed in the system configuration file located at /etc/mosquitto/mosquitto.conf.

The principal configuration parameters are summarized in Table 2.

The broker accepted client connections on TCP Port 1883. Client authentication was disabled during development, while message session persistence ensured restoration of the previous state upon system restart. Connection and message status logs were retained as file-based logs to track connectivity and address failures.

The broker categorized the received messages based on a predefined hierarchical topic structure; for example, /edv01/temp, /edv01/co2, /edv01/ammonia, and /edv01/humidity correspond to temperature, CO_2_, NH_3_, and humidity data inside the livestock housing, respectively. This structure permitted the analysis module to subscribe solely to the required information, thereby minimizing system load and enabling scalable processing.

The QoS-Level 1 (at least once) setting of MQTT was employed at sensor nodes to ensure message-transmission quality. Configured to prevent data loss, it permitted redundancy under varying network conditions. The Retain flag was disabled given the continuous streaming architecture.

Beyond message routing, the broker connects to the anomaly-detection module running on the Jetson device. The MQTT subscriber client, developed in Python (version 3.8.10), subscribes to designated topics from the Mosquitto broker continuously. Received messages are parsed, delivered to the analysis algorithm, and then logged locally in JSON or SQLite storage. This process is summarized in (Table 3).

The analysis module detects anomalies in the incoming data and converts the results into a format suitable for subsequent control stages. In this study, control-message transmission via MQTT remained unimplemented, and the control data were stored locally. This design preserves the potential for future integration with control systems while maintaining system simplicity.

**Figure 2:**
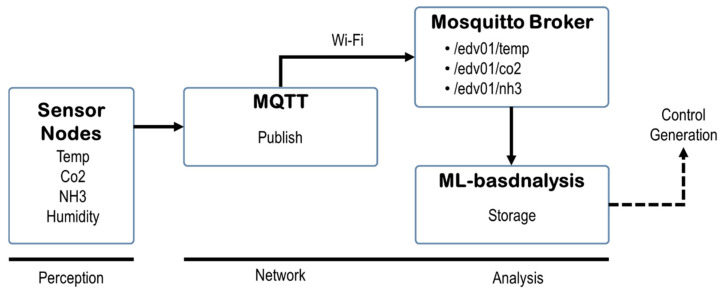
MQTT message transmission and processing flow. Solid arrows indicate actual message transmission paths, while dashed arrows represent internal processing flows.

The overall communication flow—sensor nodes publishing messages, broker routing, module processing, and result storage—is depicted in Figure 2. This configuration exemplifies edge-computing integration. This architecture ensures low-latency processing without depending on an external cloud server.

It addressed factors prevalent in livestock housing, including increasing sensor node counts, communication latency, and network instability. The experimental evaluation assessed the broker’s processing stability and data loss as node counts increased.

### 3.3. Environmental Data Collection and Preprocessing

Each sensor node integrated dedicated modules to continuously capture temperature, humidity, CO_2_, and NH_3_, given their sensitivity to environmental variations in livestock housing. The nodes used an ESP32 microcontroller (Espressif Systems, Shanghai, China), connected to DHT22 temperature–humidity sensor (Aosong Electronics, Guangzhou, China), MH-Z19B CO_2_ sensor(Winsen Electronics, Zhengzhou, China), and MQ-135 NH_3_ sensors (Winsen Electronics, Zhengzhou, China), respectively. These modules interfaced with the ESP32 via UART or analog input, converting measured values into JSON-formatted MQTT messages.

The collection cycle was five seconds, balancing sensor response times and data-processing load. This cycle aligns with the response times (2–5 s) of the MQ-135 and MH-Z19B, ensuring sufficient sensitivity for detecting environmental changes without overlapping measurements. Sensor nodes published data to the Jetson broker via WiFi-based on the designated cycle, transmitting each message to the MQTT topic path assigned to the respective sensor.

Each node packages measured data into MQTT messages in JSON format and publishes them to the Mosquitto broker installed on the Jetson device. The messages are classified into designated topics such as /edv01/temp, /edv01/co2, /dev01/ammonia, and /edv01/humidity. Thereafter, the broker delivers them to either the internal analysis module or the storage processing module.

Within the Jetson TX1, the MQTT subscriber client uses Python subscriptions to topics corresponding to each environmental sensor continuously. Upon receiving an MQTT message from a sensor node, it converts the message to structured JSON data by parsing. Each message adheres to a predefined architecture based on the measured data and is published to the broker under topics such as /edv01/temp and /edv01/co2 for the individual sensors.

The MQTT messages are constructed in JSON format, and the key items are listed in Table 4.

The Subscriber parses these items from the received messages and delivers them to the analysis module or stores them. Each parsed item serves as an input variable for the anomaly detection algorithm. To ensure analysis accuracy, preprocessing procedures, including consistency verification and missing data imputation, are applied.

1. Time synchronization is maintained by automatically correcting timestamp values that fail to match the system time based on the message reception time.
2. When data at specific time points were omitted owing to unstable WiFi-based connections or sensor errors, the last observation carried forward (LOCF) method was applied to impute missing values.
3. When output formats varied across nodes, sensor variable names were normalized using a unified schema (temperature, CO_2_, humidity, NH_3_).
4. Sensor measurements outside physically plausible ranges (e.g., CO_2_ > 10,000 ppm or NH_3_ < 0 ppm) were excluded from analysis or set to null.

The preprocessed data were used for model training and evaluation and simultaneously stored locally (JSON file or SQLite DB). Over approximately one month, more than 50,000 data points were collected. The dataset was partitioned into training, validation, and test sets based on time-series sequences. Condition-based abnormal sections—identified by criteria such as CO_2_ or NH_3_ surges and missing alive signals—accounted for approximately 12.4% of the total.

Accounting for class imbalance, precision, recall, and F1-score were utilized for model evaluation. The refined datasets were then employed to train and validate the anomaly-detection models described in Section 3.4.

### 3.4. ML Model Construction and Setting

In this study, ML-based anomaly detection models were developed to detect anomalies promptly using environmental data from livestock housing. Target anomalies comprised surges in CO_2_ or NH_3_ concentrations, abrupt fluctuations in temperature and humidity, and device non-responsiveness. Time-series data were segmented into normal and abnormal sections via manual and condition-based automatic labeling. Specific rules were defined for this labeling process. For condition-based automatic labeling, data was marked as ‘abnormal’ if CO_2_ levels exceeded 3000 ppm, NH_3_ concentrations surpassed 20 ppm, or the ‘aliveInterval’ (indicating sensor non-response) exceeded 30 s. Manual labeling was applied to identify sudden environmental spikes, such as when a sensor value increased or decreased by more than 20% compared to the previous reading.

Isolation Forest and One-Class SVM—widely used for anomaly detection—were selected for comparison. As they are highly efficient, lightweight, and common unsupervised baselines for anomaly detection in sensor data, they serve as a practical benchmark against which to evaluate the performance of the proposed temporal model (GRU); both were configured to compute outliers based on data distribution and to identify disruptions within the normal class. The input variables comprised the numerical attributes of the preprocessed environmental data, as listed in Table 5. The specific definitions for these variables, including recInterval and aliveInterval, are detailed in Table 4.

Additionally, the GRU–RNN architecture was adopted for time-series modeling, considering the low-latency processing requirement of the system. The GRU, featuring fewer parameters and a simpler computational structure than LSTM, demonstrated high inference speed and memory efficiency on edge-computing devices such as the Jetson TX1. This was confirmed in performance comparisons (detailed in Section 5.2), where the GRU (18.5 ms) achieved a significantly faster inference time than the LSTM (25.7 ms) while maintaining superior accuracy, making it the optimal candidate for edge deployment.

The GRU model comprised two hidden layers (64 units each) and a sigmoid output layer, employing ReLU and sigmoid activation functions. Adam served as the optimization algorithm with a learning rate of 0.001. A dropout rate of 0.2 mitigated overfitting.

EarlyStopping terminated training automatically based on validation loss convergence. Hyperparameters underwent grid search and manual adjustment to identify the optimal combination using validation loss.

Six input variables (temperature, humidity, CO_2_, NH_3_, recInterval, aliveInterval) were normalized within a 0–1 range. In the GRU model, a lookback window of 12 captured the time-series pattern, with each input representing sensor data from the previous minute. The dataset was split into 70% training, 15% validation, and 15% testing, and time-sequence segmentation ensured continuity.

The abnormal class accounted for ~12.4% of the total, and class_weight addressed imbalance during training. Model performance was evaluated via accuracy, precision, recall, and F1-score; low-latency capability was assessed based on inference latency in milliseconds per sample.

## 4. Experimental Setup and Evaluation Method

### 4.1. Experimental Environment and Setup

The experiment analyzed a system that effectively collected continuous livestock-housing environmental data and detected anomalies. The system comprises three components: (1) MQTT-based environmental sensor data collection, (2) ML-based anomaly-detection model training and inference, and (3) measurement of communication latency and reception rate.

The MQTT broker was operated locally by installing Mosquitto (v2.0.15) on an NVIDIA Jetson TX1, an edge device with an ARM Cortex-A57 quad-core CPU (1.9 GHz), 4 GB LPDDR4 RAM, and 16 GB eMMC storage. The device ran under the Ubuntu 20.04-based JetPack 4.6 environment.

The broker received data from sensor nodes via WiFi-based MQTT communication, and messages were delivered under topic structures such as /edv01/temp and /edv01/co2.

The WiFi-based network was established using an ASUS TUF-AX6000 access point(ASUSTeK Computer Inc., Taipei, Taiwan) operating in the 2.4 GHz band. The experiments were conducted within a 5-m radius of the access point. The average signal strength (RSSI) at the sensor nodes was consistently high (e.g., >−50 dBm), ensuring a stable connection. The environment had moderate interference from neighboring WiFi-based networks and office equipment.

We varied the QoS level (0, 1, and 2) to evaluate data reception rate, average latency, and message redundancy.

The communication port remained at the default value of 1883. Security authentication was not applied initially; the experiment was conducted with anonymous access. Messages were formatted in JSON, and multiple analysis modules concurrently subscribed to a single broker-based publish/subscribe architecture.

The hardware and software configurations of the workstation used for the machine learning experiments are detailed in Table 6.

The main libraries employed were TensorFlow 2.12, Scikit-learn 1.3.0, Pandas, and Matplotlib 3.7.2. All code was implemented in a Jupyter Notebook 6.5.4, facilitating interactive analysis and debugging.

Data in JSON format from the Jetson TX1 broker were stored locally and converted into the model training input format via Pandas-based preprocessing on the analysis server. This process relied exclusively on local files without employing external databases or cloud environments. The experiment was repeated using an identical random seed, and the results were compared using the mean and standard deviation to ensure reproducibility of model performance.

### 4.2. ML Analysis Performance Evaluation Criteria

This protocol ensured consistency across experimental iterations. Multiple metrics were employed to quantitatively evaluate the performance of the anomaly detection models based on environmental sensor data. Model effectiveness was comprehensively assessed by considering the anomaly detection omission rate (recall), excess detection rate (precision), and prompt anomaly detection accuracy. Given that anomaly detection aimed to identify problems in the greenhouse environment, binary classification was implemented, resulting in normal and anomalous outcomes.

The key performance indicators are listed in Table 7, where true positives (TP), true negatives (TN), false positives (FP), and false negatives (FN) denote the number of classifications corresponding to each predicted result.

**Table 7:** Definition of evaluation metrics.

Metric	Definition	Formula	Interpretation
Accuracy	Ratio of correctly classified samples to total samples	$\frac{\left(TP+TN\right)}{\left(TP+TN+FP+FN\right)}$ (1)	Overall correctness of the model
Precision	Ratio of true anomalies among all predicted anomalies	$\frac{\left(TP\right)}{\left(TP+FP\right)}$ (2)	How many predicted anomalies are actually true
Recall	Ratio of detected anomalies among all actual anomalies	$\frac{\left(TP\right)}{\left(TP+FN\right)}$ (3)	Model’s ability to detect real anomalies
F1-score	Harmonic mean of precision and recall	$2\;\times \;\frac{\left(Precision\times Recall\right)}{\left(Precision+Recall\right)}$ (4)	Balanced measure between precision and recall
Inference latency	Average time to produce a prediction for one sample	Measured in milliseconds/sample	Indicates low-latency feasibility on edge device

The indicators used to evaluate the low-latency processing capabilities of the Jetson TX1-based edge device are outlined in Table 8, covering the entire process from continuous data reception to anomaly judgment. These procedures collectively ensured a robust and consistent evaluation framework. The indicators were employed to assess system responsiveness and computational efficiency.

The models were evaluated using a time-series segmentation scheme comprising training (70%), validation (15%), and testing (15%) data, with these metrics repeatedly measured on the test dataset. Given that the abnormal class constituted approximately 12.4% of the data, the analysis prioritized Precision, Recall, and F1-score over accuracy.

### 4.3. System Response Time Measurement and Processing Flow

This study quantitatively examined the processing flow from data reception to anomaly detection derivation to verify low-latency feasibility in a Jetson TX1-based edge environment. Response performance was evaluated using two metrics: Inference Latency and Throughput.

- Inference Latency denotes the average processing time for the GRU model to classify a single MQTT-received sensor message as abnormal, expressed in milliseconds per sample.
- Throughput indicates the number of messages processed per second (MPS).
- Measurements were recorded for the entire process: MQTT messages were received on a Jetson TX1, parsed by a Python-based subscriber, and processed by the GRU model. The experiment was repeated for each QoS level (0, 1, and 2), and the average response time and throughput were compared across settings. In addition, the effects of communication settings on the system responsiveness of the system were evaluated through message loss and duplicate reception analysis.

The MQTT communication architecture is as follows:

1. Sensor nodes publish measurements via MQTT messages every 5 s.
2. The broker set up on the Jetson TX1 receives messages on corresponding Topics.
3. The Python-based Subscriber subscribes to these topics and parses the received messages via JSON.
4. The messages are preprocessed and fed into the GRU model for anomaly detection (0 or 1).
5. The prediction results are stored or forwarded to the subsequent control module (stored at this stage).

Visualizing this processing flow allows for intuitive identification of system flow and bottlenecks in the system flow.

## 5. Experiment Results and Analysis

This section presents an analysis of the proposed system’s performance in ML analysis and MQTT communication. The experiment was run under the environment and settings described in Section 4, with all results reported as the mean and standard deviation over ten runs.

### 5.1. Analysis of MQTT Communication Performance Results

This experiment aimed to determine the optimal QoS level in smart livestock housing by quantitatively analyzing the communication performance between a Mosquitto-based MQTT broker and sensor nodes. For this purpose, a Mosquitto broker was installed on the Jetson TX1 device, and key environmental data (e.g., temperature, humidity, and CO_2_) were published every second via WiFi-based-based sensor nodes. The sensor nodes set the message payload size at 64 bytes. The broker stored the received data continuously and subsequently delivered it to an ML-based anomaly-detection module.

The performance was evaluated using four metrics: data collection rate (%), average, maximum, and minimum latency (ms), and packet loss rate (%). Data collection rate was defined as the ratio of messages received by the broker to those published. Latency was computed as the difference between message publication time and broker reception time. Packet loss rate was defined as the ratio of packets lost during transmission. Experiments compared QoS-Level 0, QoS-Level 1, and QoS-Level 2 under identical network conditions and publication cycles. Measurements were repeated 30 times per condition to derive mean and standard deviation values. The measurement procedure followed the methodology of Mishra et al. (2021) (“Stress-Testing MQTT Brokers: A Comparative Analysis of Performance Measurements”), with message size and network conditions maintained constant to focus on broker processing performance [23].

In the experimental results, QoS Level 0 exhibited the shortest average latency (95.0 ms); nonetheless, its reliability was compromised, with the highest packet loss rate (2.10%). This outcome reflects the nature of the ‘fire-and-forget’ method, which deletes published messages without confirming the broker’s reception. In contrast, QoS Level 2 achieved the highest data collection rate (99.9%) with a packet loss rate of 0%; however, its average latency increased to 240.4 ms due to the PUBREC–PUBREL–PUBCOMP verification stages. QoS Level 1 balanced reliability and latency, achieving a stable data collection rate of 99.2 ± 0.3%, an average latency of 150.2 ± 6.4 ms, and a packet loss rate of 0.50 ± 0.2%. These findings align with the prior study that reported stability in the ARM64-based environment.

**Figure 3:**
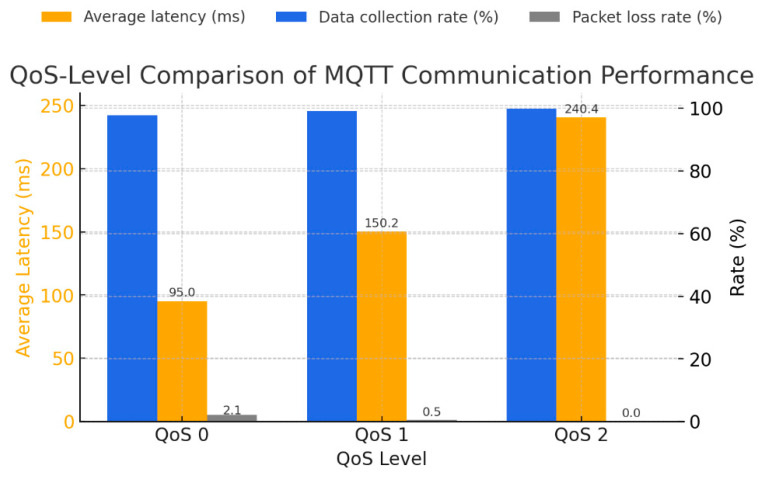
QoS-Level Comparison of MQTT Communication Performance.

The data acquisition rate, latency, and packet loss rate by QoS are summarized in Table 9 and illustrated in Figure 3.

The experimental results confirmed that data collection reliability proved more critical than low latency in livestock housing environments. Therefore, QoS Level 1 was selected as the optimal QoS level, and a balance between system responsiveness and network load was achieved by setting the message publication cycle to one second. Additionally, data loss was reduced by employing hierarchical topic naming (e.g., /edv01/env/temp and /edv01/env/humid), Retain flag activation, ensuring at least one delivery per PUBACK, and adjusting the network buffer size. The optimized QoS-Level 1 configuration achieved a data collection rate ≥ 99%, average latency ≤ 152.4 ms, and packet loss rate < 0.5%.

These results indicate that performance variations among QoS Levels 0, 1, and 2 correlate with the complexity of message retransmission and verification mechanisms. QoS Level 0 is suitable for notification services requiring minimal delay, whereas QoS Level 1 is suited for control command transmission requiring zero data loss. Thus, QoS-Level 1 is optimal for environmental monitoring and anomaly detection applications requiring a balance between these criteria. This supports this study’s choice, as it provides stable performance even in wireless communication-based smart livestock housing environments experiencing network quality degradation.

Furthermore, the impact of increasing sensor nodes on MQTT communication performance was assessed. The experimental environment was fixed at QoS 1, a message size of ≤64 bytes (environmental data in JSON format), a publication cycle of 1 s, and a measurement period of 60 s. The measurements were conducted with node counts of 1, 5, 10, 15, and 20. All nodes were connected concurrently to a single wireless network (AP) using identical configuration parameters, and all data were transmitted through a single Mosquitto broker installed on the Jetson device, ensuring synchronized data collection and timestamp alignment.

For evaluation, the proportion of messages exceeding 200 ms was included alongside average latency, data collection rate, and packet loss rate to clearly determine the point at which responsiveness (latency ≤ 200 ms) degraded.

The results demonstrated stable performance for up to ten nodes, with average latency ≤ 170 ms and a data collection rate ≥ 98%. When node counts reached 15, the average latency approached the 200 ms threshold (196.3 ± 9.2 ms), showing increased variance. Specifically, with 20 nodes, latency significantly increased to 238.7 ± 11.4 ms, and 18.2% of messages exceeded 200 ms. This degradation can be attributed to network contention (CSMA/CA collisions) and broker queue accumulation. In the QoS 1 environment, delays increased as the PUBACK response procedure accumulated ACK waiting times with multiple nodes. Therefore, when transmitting a 64-byte message every second, the system maintained stable responsiveness (latency > 200 ms) with up to 10 nodes (169.6 ± 7.5 ms), while 15 nodes represented the threshold for acceptable performance. If this range is exceeded, a publication cycle adjustment, QoS optimization, broker resource expansion, or a distributed network architecture is required.

System scalability was evaluated by analyzing performance variations as the number of sensor nodes increased. Average latency and packet loss rate were measured while increasing nodes from 1 to 20, with a 64-byte message size and a one-second publication cycle. The results demonstrated that average latency remained at ≤170 ms for up to ten nodes, approached 200 ms at 15 nodes and increased to 238.7 ms at 20 nodes. At that point, the packet loss rate exceeded 3%, indicating that approximately 15 nodes constitutes the threshold to maintain responsiveness (≤200 ms) for this configuration. The performance variations relative to node count are summarized in Table 10.

### 5.2. ML Model Performance Comparison

This section presents an analysis of the performance of the GRU-based anomaly detection model incorporating the proposed loss function compared with the Isolation Forest and One-Class SVM models. The proposed loss function integrates mean squared error (MSE) and binary cross-entropy (BCE) to minimize both regression error in time-series prediction and classification error in anomaly detection. The loss function is defined in (5).

(5) $$L=\lambda_{MSE}\cdot \frac{1}{n}\sum_{i=1}^{n}{\left(y_{i}-\hat{y_{i}}\right)}^{2}+\lambda_{BCE}\cdot \frac{-1}{n}\sum_{i=1}^{n}\left[y_{i}\log\left(\hat{y_{i}}\right)+\left(1-y_{i}\right)\log\left(1-\hat{y_{i}}\right)\right]$$

where $y_{i}$ indicates the actual label, $\hat{y_{i}}$ denotes the predicted value, and $\lambda_{MSE}$ and $\lambda_{BCE}$ represent the weights of each loss term. In this study, $\lambda_{MSE}$ = 0.6 and $\lambda_{BCE}\;$= 0.4 were set.

The model performance was evaluated using accuracy, precision, recall, and F1-score. To assess low-latency performance, inference latency (ms per sample) was measured. All models employed identical training, validation, and preprocessing conditions.

The three models are comparatively presented in Table 11. The proposed GRU achieved the highest and most stable performance across all metrics: Accuracy (97.5 ± 0.4%), Precision (0.974 ± 0.003), Recall (0.970 ± 0.004), and F1-score (0.972 ± 0.003) Furthermore, the proposed GRU model was directly compared to an LSTM-based model, which is another common temporal algorithm. As shown in Table 11, the GRU (18.5 ± 0.2 ms) demonstrated an approximately 20% faster inference speed than the LSTM (25.7 ± 0.3 ms). While the LSTM also achieved high performance (F1-score: 0.967 ± 0.004), the GRU model provided a superior balance of high accuracy and lower computational cost. Its inference latency (18.5 ± 0.2 ms) was highly consistent and met the low-latency requirements, being shorter than the One-Class SVM and only slightly longer than the Isolation Forest.

**Figure 4:**
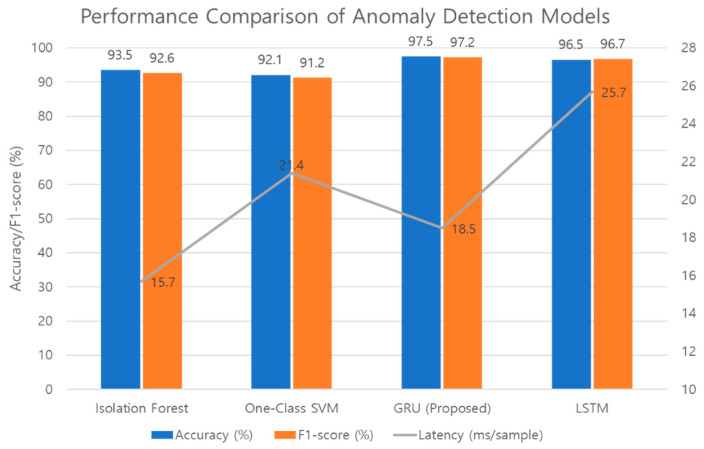
Performance Comparison of Anomaly Detection Models.

The Isolation Forest demonstrated high recall but low normal data accuracy. The One-Class SVM balanced precision and recall but did not match the GRU in overall accuracy and F1-score. The GRU performed well across all metrics, and its inference latency suited low-latency environmental applications, thus verifying the effectiveness of the proposed loss function. The comparative performance of the four models is illustrated in Figure 4.

### 5.3. Evaluation of the Integrated Performance of the System

We evaluated the integrated system combining the GRU-based anomaly detection model under the optimal communication environment (QoS-Level 1; publication cycle 1 s; message size 64 bytes; ten sensor nodes) identified in Section 5.1. The experiments ran continuously for 24 h. The network environment comprised a WiFi setting with an average bandwidth of 100 Mbps and latency ≤ 10 ms.

The performance evaluation involved four metrics: End-to-End Latency (processing time from sensor data generation to anomaly detection and storage); End-to-End Data Retention Rate (proportion of published messages stored); Processing Throughput (data samples processed per second); and System Uptime (fraction of normal service maintained during 24 h).

The evaluation yielded an average End-to-End Latency of 185.4 ± 0.2 ms, comprising MQTT transmission and broker processing (≈86%), GRU inference (10%), and data storage (4%). These results indicate that broker processing dominated delay and that network transmission and ACK handling were key bottlenecks. The Data Retention Rate reached 98.9% ± 0.3%, confirming stable data collection during operations. Most transmission failures occurred under high network load (18:00–20:00); average latency increased to 220 ms, while packet loss remained below 1%. The average Processing Throughput was 5.39 samples/s, supporting the expansion of multiple sensor nodes at a one-second publication cycle. The system update rate reached 99.6%, demonstrating that the reconnection and error recovery mechanisms ensured continuous stable operation in both the broker and analysis modules.

The performance evaluation of the integrated system is outlined in Table 12, wherein the findings are consistent with the multi-node expansion experiment described in Section 5.1. The average latency remained at 170 ms in the ten-node environment, with similar values observed within the integrated environment. Data loss occurred primarily under high network load, although packet loss remained below 1%. The inference time of the GRU model afforded parallel processing capabilities in the Jetson environment, indicating throughput remained stable across shorter publication cycles or with more sensor nodes. The network reconnection function automatically restored services after disconnections, thereby maintaining high uptime. Compared with the conventional RS485-based wired data collection method, the proposed MQTT–GRU integrated system was superior in installation flexibility, ease of maintenance, and scalability. Its data reliability permits both wireless-based scalability and a low-latency analysis function while maintaining the RS485 level.

## 6. Conclusions

In this study, an MQTT-based communication framework was designed for continuous environmental data collection with low-latency response and anomaly detection in smart livestock housing environments. Its performance was evaluated comprehensively using actual sensor data combined with ML analysis. The proposed system comprises environmental sensor nodes in the Perception Layer, a Mosquitto MQTT broker in the Network Layer, and a GRU-based anomaly detection model in the Application Layer. The end-to-end performance from data generation to storage was evaluated via interlayer linkage.

In the MQTT broker performance evaluation, latency and packet loss rates varied with the QoS level. The QoS-Level 1 setting exhibited the most stable transmission performance, with an average latency of approximately 150.2 ± 6.4 ms, a data collection rate ≥ 99%, and a packet loss rate ≤ 0.5%. In an experiment increasing the number of sensor nodes, system responsiveness (latency ≤ 200 ms) was maintained for 10 to 15 nodes with a message size of 64 bytes, whereas the average latency increased to 238.7 ± 11.4 ms and the packet loss rate to ≥3% for 20 nodes.

To compare ML-model performance, the Isolation Forest, One-Class SVM, and the proposed GRU were applied to the same dataset. The proposed GRU model achieved consistently high accuracy and low latency: 97.5 ± 0.4% accuracy, an F1-score of 0.972 ± 0.003, and an inference time of 18.5 ± 0.2 ms/sample. These results indicate the model effectively reduces system response time and enables prompt anomaly detection.

In the integrated experiment, the mean End-to-End Latency was 185.4 ± 0.2 ms, the Data Retention Rate 98.9 ± 0.3%, the Processing Throughput 5.39 samples/s, and the System Uptime 99.6% in a QoS-Level 1 environment comprising ten sensor nodes. These findings confirm that the optimal conditions identified in the individual communication and model evaluations remained stable in the integrated operating environment.

Compared to conventional RS485-based wired data collection, the proposed MQTT-based system eliminates wiring, enables facile installation and rearrangement, and offers high node scalability via wireless communication. Compared to the HTTP-based transmission method, system responsiveness improved under equivalent message-size conditions: the average latency decreased by approximately 30–40%, owing to ACK-based QoS control and the lightweight protocol architecture.

However, this study did not include an integrated demonstration with an actual controller; the experiments focused solely on environmental data from sensor nodes. Future research should focus on implementing the closed-loop control logic. This involves designing a control-side MQTT subscriber (e.g., on an ESP32-based actuator) that subscribes to dedicated ‘anomaly/control’ topics. This would enable the system to autonomously execute corrective actions, such as activating ventilation fans, heaters, or alarm systems, transforming it from a passive monitoring system into an active environmental management solution.

Additionally, exploring deployment on lower-cost embedded systems (e.g., high-performance ESP or Raspberry Pi boards) and incorporating Real-Time Operating Systems (RTOS) to pursue stricter real-time guarantees would be valuable extensions. Further research could also involve comparative analysis with other temporal models, such as Convolutional Neural Networks (CNNs) or Temporal Convolutional Networks (TCNs), to explore different architectures for anomaly detection.

Nevertheless, this study provided integrated verification of the MQTT communication design and the ML analysis using actual environmental data. This study is significant because it simultaneously assesses sensor node scalability, system responsiveness, and system stability. Notably, the QoS-Level 1-based optimal communication setting and lightweight GRU model architecture apply to various IoT-based agricultural and livestock environment monitoring systems and smart livestock housing.

## Figures and Tables

**Table 1:** Comparison of communication protocols for livestock housing environments.

Feature	RS-485	Modbus RTU	HTTP (WiFi-Based)	MQTT
Physical Layer	Wired (Serial)	Wired (RS-485 based)	Wireless (WiFi-based)	Wired or Wireless
Scalability	Low	Moderate	High	High (Broker-based)
Responsiveness/Latency	Medium	Low	Low	High (Low-latency)
Maintenance Effort	High (cabling)	Moderate	Moderate	Low (lightweight)
Communication Pattern	Master–Slave	Master–Slave (polling)	Client–Server	Publish/Subscribe
Suitability for Piggery	Low	Moderate	Moderate	High

Note: The experiment was conducted in a controlled local WiFi-based environment with a message size of 64 bytes and a publishing interval of one second. Latency values represent the time from message publication to broker reception.

**Table 2:** Summary of MQTT broker configuration settings.

Configuration Item	Value	Description
listener	1883	Default TCP port for broker
persistence	true	Enables message/Session state storage
persistence_location	/var/lib/mosquitto/	Path for persisted data
log_dest	file	Logs written to file
log_type	error, notice, warning	Log categories for monitoring
connection_messages	true	Enables logging of client connect/disconnect
allow_anonymous	true	Allows for unauthenticated access (for development)

Note: Each node transmitted environmental data in JSON format (64 bytes) at one message per second for 60 s. All nodes connected to a single WiFi-based access point and communicated with a Mosquitto broker deployed on Jetson.

**Table 3:** Data interface between MQTT broker and anomaly detection module.

Field	Description
Communication Type	MQTT Subscribe (broker to analysis module)
Subscription Method	Python-based client using paho-mqtt
Subscribed Topics	/edv01/temp, /edv01/co2, /edv01/humidity, /edv01/ammonia
Message Format	JSON (includes device ID, timestamp, measurement, interval)
Processing Logic	JSON parsing → model input transformation
Result Handling	Stored locally as JSON or in SQLite
Control Feedback	Not implemented; control data is generated and saved only

Note: The interface was implemented in Python using paho-mqtt library, with subscription to the broker topics defined in Section 3.2.

**Table 4:** Structure of MQTT JSON payload received from sensor nodes.

Field	Type	Description
deviceId	String	Unique identifier of the sensor node (e.g., MAC or internal ID)
timestamp	Integer	Unix time (UTC) when the data was recorded
temperature	Float	Measured ambient temperature (°C)
humidity	Float	Measured relative humidity (%)
co2	Float	Measured carbon dioxide level (ppm)
nh3	Float	Measured ammonia concentration (ppm)
recInterval	Integer	Data recording interval in seconds
aliveInterval	Integer	Heartbeat interval to verify device status

**Table 5:** Initial configuration of anomaly detection models.

Parameter	Isolation Forest	One-Class SVM	GRU (Proposed)
Model Type	Tree-based Ensemble	Kernel-based Classifier	Recurrent Neural Network (GRU)
Input Features	Temp, Humidity, CO_2_, NH_3_, recInterval, aliveInterval	Same	Lookback window × feature matrix
Normalization	Min-Max scaling	Min-Max scaling	Min-Max scaling
Time Context	None	None	Lookback window (e.g., 12 steps)
Contamination Ratio	0.12	0.12	—
Kernel/Criterion	—	RBF kernel	ReLU (hidden), Sigmoid (output)
Hidden Layers	—	—	2 GRU layers (64 units each)
Optimizer	—	—	Adam (lr = 0.001)
Regularization	—	ν = 0.05	Dropout = 0.2
Output Type	Anomaly Score	Binary (normal/anomaly)	Binary (0 or 1)
Parameter	Isolation Forest	One-Class SVM	GRU (Proposed)
Model Type	Tree-based Ensemble	Kernel-based Classifier	Recurrent Neural Network (GRU)
Input Features	Temp, Humidity, CO_2_, NH_3_, recInterval, aliveInterval	Same	Lookback window × feature matrix

**Table 6:** Summary of experimental environment.

Category	Configuration
Edge Device (Broker)	NVIDIA Jetson TX1 CPU: ARM Cortex-A57 (1.9 GHz × 4) RAM: 4 GB LPDDR4 Storage: 16 GB eMMC OS: Ubuntu 20.04 MQTT Broker: Mosquitto 2.0.15
MQTT Communication	WiFi-based based Port: 1883 QoS levels: 0/1/2 (all tested) Authentication: Anonymous (for development stage)
Broker Structure	Single broker with multiple subscribers Topic structure: /edv01/temp, /edv01/co2, etc.
ML Training System	Intel Core i7-10700 (2.9 GHz) RAM: 32 GB GPU: NVIDIA GTX 1660 OS: Ubuntu 20.04
Software Stack	Python 3.9 (conda environment) TensorFlow 2.12 Scikit-learn 1.3.0 Pandas, Matplotlib 3.7.2
Data Processing	JSON to Pandas pre-processing Local file-based pipeline (no external DB)

Note: All performance evaluations were conducted under these hardware and software configurations unless otherwise stated.

**Table 8:** System responsiveness metrics.

Metric	Unit	Definition	Interpretation
Inference latency	milliseconds/sample	Average time required to predict anomaly for a single input sample	Indicates how fast the model can respond in real-time
Throughput	messages/second (MPS)	Number of messages that can be processed per second by the model	Indicates the model’s processing capacity per second

**Table 9:** Performance of MQTT communication under different QoS levels.

QoS Level	Data Collection Rate (%)	Avg. Latency (ms)	Max. Latency (ms)	Min. Latency (ms)	Packet Loss Rate (%)
QoS 0	97.8 ± 0.6	95.0 ± 5.2	132.0 ± 7.0	80.0 ± 04.5	2.10 ± 0.5
QoS 1	99.2 ± 0.3	150.2 ± 6.4	185.0 ± 8.5	140.0 ± 5.0	0.50 ± 0.2
QoS 2	99.9 ± 0.6	240.4 ± 8.1	310.0 ± 10.5	220.0 ± 7.0	0.00 ± 0.0

Note: Experiment conducted over a local WiFi-based network; message size, 64 bytes; publishing interval, 1 s; and 10 active sensor nodes.

**Table 10:** Performance variation by the number of sensor nodes (Mean ± SD over 10 runs).

Number of Sensor Nodes	Avg. Latency (ms)	Data Collection Rate (%)	Packet Loss Rate (%)	Latency > 200 ms (%)	Total Data Sent (KB/min)
1	152.0 ± 5.8	99.2 ± 0.3	0.5 ± 0.2	0.3 ± 0.1	64
5	160.8 ± 6.1	99.0 ± 0.3	0.6 ± 0.3	0.5 ± 0.2	320
10	169.6 ± 7.5	98.8 ± 0.4	0.8 ± 0.4	0.9 ± 0.3	640
15	196.3 ± 9.2	98.1 ± 0.7	1.2 ± 0.7	6.4 ± 1.5	960
20	238.7 ± 11.4	96.8 ± 1.1	3.1 ± 1.1	18.2 ± 2.5	1280

Note: Each node transmitted 64 bytes JSON-formatted environmental data at 1 Hz for 60 s to a single Mosquitto broker over WiFi-based.

**Table 11:** Performance comparison of anomaly detection models (Mean ± SD over 10 runs).

Model	Accuracy (%)	Precision	Recall	F1-Score	Inference Time (ms/Sample)
Isolation Forest	93.5 ± 0.8	0.914 ± 0.007	0.938 ± 0.005	0.926 ± 0.006	15.7 ± 0.3
One-Class SVM	92.1 ± 1.0	0.901 ± 0.009	0.923 ± 0.008	0.912 ± 0.008	21.4 ± 0.4
GRU (Proposed)	97.5 ± 0.4	0.974 ± 0.003	0.970 ± 0.004	0.972 ± 0.003	18.5 ± 0.2
LSTM	96.5 ± 0.5	0.968 ± 0.004	0.965 ± 0.005	0.967 ± 0.004	25.7 ± 0.3

**Table 12:** Integrated performance metrics of the proposed smart livestock anomaly detection system (Mean ± SD over 10 runs).

Metric	Measured Value (Mean ± SD)
End-to-End Latency (ms)	185.4 ± 0.2
End-to-End Data Retention Rate (%)	98.9 ± 0.3
Processing Throughput (samples/s)	5.39 ± 0.15
System Uptime (%)	99.6 ± 0.1

## Data Availability

The data presented in this study are not publicly available due to operational restrictions but are available from the corresponding author upon reasonable request.
